# Низкорослость, обусловленная задержкой внутриутробного развития. Клинические и гормонально-метаболические особенности, возможности ростостимулирующей терапии

**DOI:** 10.14341/probl13178

**Published:** 2022-10-23

**Authors:** Е. В. Нагаева

**Affiliations:** Национальный медицинский исследовательский центр эндокринологии

**Keywords:** низкорослость, дети, задержка внутриутробного развития, рост, гормон роста, инсулиноподобный фактор роста, метаболический синдром, соматропин, терапия гормоном роста

## Abstract

В статье представлены данные о низкорослости, обусловленной задержкой внутриутробного развития. Данный вид низкорослости, выделенный в отдельную нозологию, объединяет детей, родившихся с малыми относительно срока беременности параметрами длины и массы тела. У подавляющего большинства из них в первые годы жизни наблюдаются ускоренные темпы роста, позволяющие ребенку нормализовать свои весоростовые показатели и догнать в развитии сверстников. В случае отсутствия постнатального ростового скачка дети имеют высокий риск на протяжении всего детства отставать в физическом развитии, достигнуть низкого конечного роста и стать низкорослыми взрослыми. Помимо этого, факт рождения с малыми размерами тела ассоциирован с рядом гормонально-метаболических особенностей, отдаленным риском развития метаболического синдрома во взрослые годы.

Предполагается, что отсутствие постнатального ростового ускорения обусловлено различными повреждениями оси соматотропный гормон/инсулиноподобный фактор роста 1-го типа (СТГ-ИФР1) — парциальным дефицитом СТГ, парциальной резистентностью к СТГ, парциальной резистентностью к ИФР1. Ростостимулирующая терапия гормоном роста, начатая в раннем возрасте, способна нормализовать его темпы в детстве и в конечном итоге значительно улучшить или нормализовать конечный рост низкорослых детей, имевших задержку внутриутробного развития в анамнезе.

## Abstract (en)

The article presents data about short stature due to intrauterine development delay. This type of short stature — separate nosology, unites children born small for gestation age. The majority of them in the first years of life have accelerated growth rates, allowing the child to normalize their weight-growth indicators and catch up in the development of peers. In the absence of an accelerated growth rates, children have a high risk of lagging behind in physical development throughout childhood, achieving low final growth and becoming short adults. In addition, the fact of birth with small body sizes is associated with a number of hormonal and metabolic features, a risk of metabolic syndrome in adult years.

It is assumed that the absence of postnatal growth acceleration is due to various damages to the GH-IGF1 axis (partial GH deficiency, partial resistance to GH, partial resistance to IGF1). Growth hormone therapy, initiated early in life, is able to normalize growth rates in childhood and ultimately significantly improve or normalize the final growth of short stature children born small for gestational age.

## ОПРЕДЕЛЕНИЕ

Термин «small for gestational age» (SGA) применяется в отношении детей, рожденных с низкими параметрами тела (масса и/или длина тела) — рожденных маленькими относительно гестационного возраста (МОГВ). Такие дети могут быть как доношенными, так и недоношенными.

В разное время в разных странах, а также у врачей различных специальностей в качестве нижней границы нормы размеров тела при рождении использовались различные критерии. В 2001 г. был достигнут международный консенсус в понимании того, кто же относится к категории МОГВ: это новорожденные (доношенные и недоношенные), чья масса и/или длина тела при рождении более чем на 2 SD ниже средних референсных значений (или ниже 3-го перцентиля) для соответствующего срока беременности [1][2].

Термин МОГВ зачастую используют как синоним задержки внутриутробного развития (ЗВУР), однако абсолютно эквивалентными эти определения не являются. ЗВУР отражает факт снижения скорости внутриутробного роста, который можно выявить посредством как минимум двух ультразвуковых измерений размеров плода, сделанных на разных сроках гестации [1]. В свою очередь, МОГВ — это размер тела ребенка при рождении без указания темпов его внутриутробного роста. Задержка внутриутробного роста не всегда приводит к МОГВ, однако в подавляющем большинстве случаев МОГВ является следствием ЗВУР. В Российской Федерации нет общепринятого перевода SGA, для обозначения таких детей по-прежнему используется более употребимый термин ЗВУР.

## ЭПИДЕМИОЛОГИЯ

По данным ранних популяционных исследований, примерно 5% детей соответствовали критериям МОГВ, при этом в последние годы наблюдается тенденция к их увеличению: доля таких детей в настоящее время соответствует 16% [3]. Частота рождения МОГВ-детей в значительной степени зависит от социального уровня жизни общества, колеблясь от 7% в промышленно развитых государствах до 41,5% — в странах с низким экономическим статусом [4][5]. В 2010 г. в странах с низким и средним доходом на душу населения родились 32 400 000 МОГВ-детей, что составило 27% всех живорожденных, при этом подавляющее большинство из них — 29 700 000 были доношенными [4]. Считается, что среди всех низкорослых взрослых 20% — это люди, родившиеся МОГВ [3].

Применение различных референсных стандартов в определении МОГВ-детей приводит к выраженным колебаниям их распространенности даже в пределах одной отдельно взятой страны, например, от 10,5 до 72,5% (Непал), от 12,0 до 78,4% (Индия) [6–8]. Поскольку предложенная стандартизация параметров тела при рождении принята далеко не всеми странами, истинная распространенность МОГВ до сих пор остается неизвестной [9][10].

## ЭТИОЛОГИЯ МОГВ/ЗВУР

Для нормального роста и развития плода крайне важен баланс между экзогенными и эндогенными факторами. Любые причины, приводящие к недостаточному поступлению питательных веществ и кислорода (недоедание, несбалансированное питание матери, аномалии сосудов плаценты или нарушения ее функции), могут привести к снижению питания плода и задержке его развития [11][12], преимущественно на поздних сроках беременности [13][14]. Около 20–30% случаев ЗВУР обусловлено эндогенными факторами, в частности генетическими аномалиями плода [15] и синдромальной патологией, например синдромами Сильвера–Рассела, Секкеля, Шерешевского–Тернера, Дауна, Вильямса и др.

Сопутствующие заболевания матери, в первую очередь артериальная гипертензия, на долю которой приходится до 1/3 всех случаев ЗВУР [16][17]; заболевания почек; инфекции, перенесенные во время беременности (в особенности краснуха, токсоплазмоз и ВИЧ); неадекватное питание; вредные привычки (алкогольная, никотиновая, наркотическая зависимости) и прием не разрешенных во время беременности препаратов [14] составляют материнскую группу причин ЗВУР. Курение во время беременности повышает риск ЗВУР в 3 раза, суммарно увеличивая его до 18% [18][19].

Большой или слишком юный возраст матери также может влиять на размеры новорожденного ребенка. При наличии обоих родителей, имевших малый размер тела при рождении, риск МОГВ у их детей повышен более чем в 16 раз [20].

Наиболее значимым прогностическим фактором массы тела ребенка при рождении является прибавка массы тела матери во время беременности (ее разница между 3–5-м и 9-м месяцами гестации). Показано, что увеличение массы тела матери на 1 кг ассоциировано с увеличением массы тела ребенка при рождении на 260 г, наличие избыточного веса или ожирения у матери до зачатия — с повышенным риском макросомии, перинатальной смертности, с одной стороны, и со сниженным риском ЗВУР — с другой [21].

Повышен риск появления МОГВ-детей и при многоплодной беременности [22], плацентарной недостаточности или аномалиях плаценты, когда возникает диссонанс между возможностями плацентарной перфузии и потребностями плода в адекватной оксигенации [23].

В серии экспериментов на мышах было показано, что инактивация генов, кодирующих инсулин, инсулиноподобные факторы роста (ИФР) -1 и -2, а также их специфические рецепторы приводила к развитию тяжелой ЗВУР и низкой постнатальной скорости роста, свидетельствуя, что МОГВ и ЗВУР могут быть результатом нарушения инсулиновой и ИФР-активности во время внутриутробного развития плода [24]. Мутации в гене ИФР1 были обнаружены и у детей с ЗВУР и выраженной постнатальной задержкой роста [25].

Существуют свидетельства того, что на массу тела при рождении может влиять расовое и этническое происхождение: риск ЗВУР плода выше у представительниц монголоидной и негроидной рас, при этом нельзя полностью исключить вклад социально-экономических факторов. Так, при анализе массы тела новорожденных от американок европеоидной и негроидной рас оказалось, что влияние социально-экономических факторов на риск рождения маловесных детей и перинатальную смертность значимо превалирует над таковым генетических [26].

## РОСТ ПЛОДА И ГОРМОНАЛЬНАЯ РЕГУЛЯЦИЯ

Известно, что важнейшими факторами, вовлеченными в регуляцию фетального и неонатального роста, являются ИФР1, ИФР2, ИФР-связывающий белок 1 (ИФРСБ1), ИФР-связывающий белок 3 (ИФРСБ3) и инсулин [27][28], уровни которых в сыворотке плода возрастают по мере увеличения срока гестации и зависят от поступления к нему питательных веществ. Полагают, что на поздних сроках беременности к ключевым гормонам, контролирующим рост плода, относятся ИФР1 и -2 [28][29], главным регулятором продукции которых, в отличие от постнатального периода, считается инсулин, а не соматотропный гормон (СТГ) [29].

В ряде исследований было обнаружено, что концентрации инсулина, ИФР1, -2 и -СБ3 в пуповинной крови у маловесных детей непосредственно после рождения ниже, тогда как уровни ИФРСБ1 и СТГ — значительно выше соответствующих показателей детей с нормальными и большими весоростовыми показателями при рождении [30]. После рождения у большинства детей с ЗВУР наблюдается увеличение концентрации ИФР1, что свидетельствует об активации функционирования соматотропной оси — механизма, лежащего в основе догоняющих темпов роста [30].

Развитие жировой ткани и набор жировой массы в значительной степени обусловлены уровнями фетального лептина [31], определяемого у плода с 18-й недели развития. Для детей с ЗВУР характерны низкие концентрации лептина, сочетающиеся снизкой массой жировой ткани [32].

## РОСТ У ДЕТЕЙ С ЗАДЕРЖКОЙ ВНУТРИУТРОБНОГО РАЗВИТИЯ

Популяционные исследования показали, что около 8–10% детей, рожденных с ЗВУР, остаются низкорослыми (SDS роста <-2) на протяжении всего детства по причине отсутствия или недостаточной выраженности постнатального ускорения темпов роста [33][34]. Конечный рост при этом в среднем на 1 SD меньше генетически прогнозируемого [3][35]. Неудовлетворительные результаты конечного роста отчасти обусловлены отсутствием пубертатного ростового скачка, в связи с чем SDS конечного роста МОГВ-детей практически не отличается от их SDS роста в допубертатные годы [32].

Под постнатальным ростовым скачком понимают высокие «догоняющие» темпы роста, наблюдающиеся в течение первые несколько месяцев/лет жизни, позволяющие детям нормализовать свой рост [3][36]. Karlberg J. и Albertsson-Wikland K. еще в1995 г. опубликовали большое популяционное исследование, в котором 3650 здоровых доношенных шведских детей наблюдались в возрасте от рождения до 18 лет [3]. К 1 году жизни 13% детей, родившихся МОГВ, имели рост менее -2 SD, к 18 годам остались низкорослыми (рост <-2 SD) 8% наблюдавшихся. Средний конечный рост тех, кто был низкорослым в 2-летнем возрасте, оказался на 1,84 SD ниже (на 12,1 см) по сравнению с теми, кто в 2-летнем возрасте не отставал в росте [3]. Взрослые, имеющие ЗВУР в анамнезе, составляют примерно 22% общей популяции низкорослых совершеннолетних людей [3]. В более малочисленном исследовании от рождения до достижения конечного роста (средний возраст 21±2 года) наблюдались 213 детей с ЗВУР (группа 1) и 272 с нормальными весоростовыми параметрами тела при рождении (группа 2). Обнаружено, что низкорослыми взрослыми (SDS роста <-2) в 1-й группе стали 13,6% по сравнению с 1,7% — во 2-й [37].

Существует положительная корреляция между выраженностью постнатального ростового скачка, весоростовыми показателями при рождении, с одной стороны, и целевым ростом — с другой [3]. В случае рождения с низкой длиной тела риск стать низкорослым взрослым в 7 раз, при рождении с малой массой — в 5 раз выше по сравнению со сверстниками, родившимися с нормальными параметрами тела [3]. Помимо этого, у детей с ЗВУР важнейшими прогностическими факторами достижения неудовлетворительного конечного роста являются низкие весоростовые показатели на момент начала пубертата [3][32][35]. Влияние генетического потенциала также существенно: риск стать низкорослым совершеннолетним значительно возрастает при наличии низкорослых родителей [3].

В России конечный рост низкорослых детей с ЗВУР значительно меньше их генетически прогнозируемого и составляет у мальчиков 158,0±3,9 см (медиана SDS -2,5 [ -4,7÷-1,3]) против 174,0±4,6 см (медиана SDS -0,1 [ -1,5÷1,1]); у девочек — 143,1±4,7 см (медиана SDS -3,0 [ -4,6÷-2,3]) против 159,4±5,9 см (медиана SDS -0,3 [ -2,7÷1,7]) соответственно [38]. Таким образом, мальчики не дорастают до роста своих биологических родителей в среднем на 16,0±4,3 см, девочки — на 16,4±5,0 см, дельта SDS вне зависимости от гендерной принадлежности составляет 2,5.

## ГОРМОНАЛЬНЫЙ СТАТУС НИЗКОРОСЛЫХ ДЕТЕЙ С ЗВУР

Патофизиология неадекватного постнатального ускорения роста у низкорослых детей с ЗВУР изучена крайне недостаточно, кроме того, она может быть различной, поскольку группа низкорослых детей с ЗВУР сама по себе гетерогенна. Предполагается, что основой низкой амплитуды ростового скачка у детей с ЗВУР являются нарушения активности СТГ, ИФР1 и инсулина [32][39][40], которые, как полагают, могут иметь до 60% низкорослых детей с ЗВУР [39].

Для некоторых низкорослых детей с ЗВУР характерны низкие стимулированные уровни СТГ или низкая спонтанная секреция СТГ при сниженных концентрациях ИФР1 и ИФРСБ3, что свидетельствует о наличии дефицита СТГ; другие имеют нормальную максимально стимулированную концентрацию СТГ, но сниженные уровни ИФР1 и ИФРСБ3, что предполагает наличие сниженной чувствительности к СТГ [39]. Рассматривается и вероятность сниженной чувствительности к ИФР1, о чем говорят наблюдаемые в ряде случаев высоко-нормальные концентрации СТГ и ИФР1 [39]. Не исключена и возможность сочетания вышеописанных повреждений [38][41]. Различные аномалии СТГ-ИФР-оси, вероятно, являются отражением различных причин задержки внутриутробного роста.

## ОСОБЕННОСТИ НИЗКОРОСЛЫХ ДЕТЕЙ С ЗВУР

Дефицит массы тела, являющийся типичной особенностью низкорослых детей с ЗВУР допубертатного возраста [38][42], может быть следствием проблем питания, вызванных резко сниженным аппетитом в первые годы жизни [38].

Степень костного созревания, отрицательно коррелируя с ростовым потенциалом, имеет крайне важное значение в прогнозировании конечного роста. У низкорослых детей с ЗВУР младшего возраста скорость костного созревания, как правило, замедлена, а отставание от хронологического возраста может достигать 3–4 лет. По мере взросления ребенка наблюдаются спонтанная прогрессия костного возраста и нивелирование его отставания к моменту начала полового развития, при этом задержка в росте сохраняется [38][43].

## ПОЛОВОЕ РАЗВИТИЕ НИЗКОРОСЛЫХ ДЕТЕЙ С ЗВУР

Некоторые авторы свидетельствуют о том, что большинство низкорослых детей с ЗВУР вступают в период полового развития в нормальные для общей популяции сроки [44]. Другие отмечают у данной категории детей склонность к ускоренному половому развитию, уменьшению его продолжительности, снижению амплитуды пубертатного ростового скачка или его отсутствию [32], что способствует достижению неудовлетворительного конечного роста [32][38][45]. Вместе с тем изменения времени начала пубертата у детей с ЗВУР, вероятно, обусловлены не только самим фактом рождения с низкими параметрами тела, но и другими, включая этническую принадлежность, особенности питания.

Метаанализ 20 исследований выявил более раннее начало полового развития у детей с ЗВУР, особенно лиц женского пола: более раннее наступление менархе [46]. С другой стороны, показано, что наступление преждевременного адренархе более характерно для детей с выраженными постнатальными темпами роста и набора массы тела [43][47].

## РИСК РАЗВИТИЯ МЕТАБОЛИЧЕСКОГО СИНДРОМА И ЗВУР

Эпидемиологические исследования продемонстрировали, что люди с ЗВУР в зрелые годы жизни имеют повышенный риск развития метаболического синдрома. На сегодняшний день большинством авторов признается факт, что такая ассоциация в большей степени характерна для тех, у кого в постнатальном периоде наблюдалась быстрая прибавка массы тела [48]. Показано, что низкие весоростовые показатели при рождении являются независимым фактором повышенного риска развития инсулиновой резистентности во второй половине жизни человека [35]. Маловесные дети имеют более высокие концентрации инсулина натощак и сниженную чувствительность к инсулину по сравнению с низкорослыми сверстниками, родившимися с нормальными параметрами тела [40]. Вместе с тем все больше данных свидетельствуют о том, что развитие инсулиновой резистентности у маловесных детей связано не столько с фактом ЗВУР, сколько с избыточным весом/ожирением: дети с быстрым нарастанием массы тела демонстрируют низкую чувствительность к инсулину, при этом развитие инсулиновой резистентности не связано с выраженностью постнатального ускорения в росте [49].

Несмотря на множество опубликованных работ, на сегодняшний день нет убедительных доказательств наличия у детей с ЗВУР по сравнению со сверстниками, родившимися с нормальными параметрами тела, более высокой частоты встречаемости сахарного диабета 2 типа, нарушенной толерантности к глюкозе, дислипидемии или артериальной гипертензии [50]. Метаболический синдром, повышенный риск развития которого отмечается у лиц, имевших ЗВУР в анамнезе, как правило, дебютирует в зрелые годы (в возрасте старше 22 лет). У детей, родившихся МОГВ, как и в общей популяции, быстрый набор массы тела, избыточная масса тела или ожирение, вероятно, являются главными определяющими факторами будущих метаболических проблем. Помимо этого, любой риск развития метаболических нарушений, связанный с ЗВУР, может быть усилен присутствием других факторов риска, таких как отягощенная наследственность, этническая принадлежность и др. [51][52].

## ПСИХОЛОГИЧЕСКИЕ ОСОБЕННОСТИ ДЕТЕЙ С ЗВУР

По сравнению со сверстниками, родившимися с нормальными параметрами тела, дети с ЗВУР отличаются более высоким уровнем поведенческих проблем и дефицита внимания, более низким уровнем достижений, знаний и навыков [53]. Факт рождения с низкой длиной и/или массой тела ассоциирован с повышенным риском субнормального интеллектуального, психологического развития и гиперактивности, приводящим к более скромным школьным достижениям. Риск возникновения психосоциальных проблем выше у детей, не имевших спонтанного ростового скачка в раннем детстве, что, безусловно, вызвано негативным вкладом низкорослости.

В настоящее время ведется полемика о роли метаболизма глюкозы, инсулина, ИФР1 и -2 на развитие центральной нервной системы и когнитивную функцию ребенка [54]. Помимо этого, такие факторы риска возникновения ЗВУР, как неадекватное питание, вредные привычки матери, влияют на плод, вероятно, снижая концентрацию глюкозы в головном мозге и воздействуя на функцию ИФР [55], что теоретически может объяснить связь между изменениями когнитивной функции и малыми размерами тела при рождении.

В современном мире существует негативное отношение к низкорослым людям. Имея, помимо отставания в росте, значительный дефицит массы тела, низкорослые дети с ЗВУР отличаются от сверстников сниженной самооценкой, что зачастую является причиной появления чувства неуверенности, излишней ранимости и невысокого социального положения в обществе. Длительное наблюдение за взрослыми людьми с ЗВУР в анамнезе не выявило различий по уровню занятости и семейного положения по сравнению с людьми, родившимися с нормальными параметрами тела, тем не менее, они занимают более низкие профессиональные позиции и имеют значительно меньший материальный доход [56].

## ТЕРАПИЯ СОМАТРОПИНОМ У НИЗКОРОСЛЫХ ДЕТЕЙ С ЗВУР

Первые попытки лечения низкорослых детей с ЗВУР были предприняты почти 40 лет назад. Официальное одобрение Управлением по контролю за продуктами и лекарствами США (FDA, Food and Drug Administration) применения соматропина при низкорослости, обусловленной ЗВУР, относится к 2001 г., чуть позднее — в 2003 г. решение об одобрении вынесло Европейское агентство оценки лекарственных препаратов (European Agency for the Evaluation of Medicinal Products, EMEA) (табл. 1).

**Table:** Таблица 1. Одобренные показания для применения соматропина у низкорослых детей с ЗВУРTable 1. Approved indications for the use of somatropin in small children with intrauterine growth retardation

Показатели на момент начала терапии	FDA 2001	EMEA 2003
Минимальный хронологический возраст, годы	2	4
SDS роста	Не определено	-2,5 SD
Скорость роста	Отсутствие ростового скачка	<0 SD для соответствующего возраста
SDS роста относительно SDS средне-родительского роста	Не определен	SDS роста более чем на 1 SD ниже SDS средне-родительского роста
Доза соматропина, мкг/кг/день	70	35

Широкий спектр изменений оси СТГ-ИФР1, указывающий, вероятно, на разнородность механизмов низкорослости у детей с ЗВУР [38][57], не прогнозирует сценарий динамики роста в детстве, в связи с чем было высказано предположение о том, что экзогенное введение соматропина у таких детей способно индуцировать ростовой скачок посредством преодоления вероятно имеющейся/ихся резистентности/ей внутри системы СТГ-ИФР.

Ростовой ответ на терапию соматропином у низкорослых детей с ЗВУР может быть схож с ответом детей как с дефицитом гормона роста (ДГР), так и без него [58][59], в связи с чем терапия соматропином у данных детей может рассматриваться вне зависимости от СТГ-статуса, определяемого посредством проведения СТГ-стимуляционных тестов.

Целями лечения детей с ЗВУР гормоном роста (ГР), как и лечения детей с иными вариантами низкорослости, являются нормализация роста, поддержание нормальных темпов роста в детстве и достижение конечного роста в пределах нормальных популяционных значений.

Оптимальные сроки начала терапии соматропином. Вопрос о начале лечения ГР у низкорослых детей с ЗВУР может рассматриваться исключительно после истечения времени развития спонтанного постнатального ускорения в росте, обычно после 2–3-летнего возраста, у глубоконедоношенных детей — возможно, и в более поздние сроки [60]. Как и при ДГР, эффективность терапии напрямую зависит от ее длительности [61].

Результаты многолетнего применения ГР. Одно из первых крупных многоцентровых рандомизированных исследований длительного применения ГР было проведено в 1990 годы. Под наблюдением находились 79 низкорослых детей с ЗВУР допубертатного возраста [59]. Пациенты (мужского пола в возрасте 3–11 лет, женского — 3–9 лет) с и без нарушений в системе СТГ-ИФР были рандомизированы в одну из двух дозовых групп (0,033 или 0,067 мг/кг/сут). Критериями включения были: рост при рождении <3‰ (SDS <-1,88) для соответствующего срока гестации и недостаточный постнатальный ростовой скачок (рост <3‰ в течение первых 2 лет жизни и позднее, скорость роста <50‰ для соответствующего хронологического возраста (ХВ). Все дети имели допубертатный статус. Терапия соматропином была непрерывной и длилась до достижения конечного или околоконечного роста (скорость роста <0,5–2 см за 6 мес). Исходно по антропометрическим показателям группы различий не имели, контроля без лечения в исследовании не было [59]. Средний SDS роста при рождении составил -3,65, что оказалось значительно ниже требуемого значения (<-1,88). Несмотря на критерий включения (SDS роста <1,88 для соответствующего ХВ и пола), дети имели выраженное отставание в росте: SDS роста на момент включения: -3,0. ДГР не входил в критерии исключения, тем не менее максимальное стимулированное значение СТГ<10,0 нг/мл было получено у 27 (34%) из 79 детей.

За 5 лет терапии SDS роста значительно увеличился по сравнению с базальным значением, практически каждый леченый ребенок достиг роста в пределах здоровой детской популяции, средний SDS роста в обеих группах стал соответствовать диапазону SDS средне-родительского роста [59]. Дозозависимый эффект терапии наблюдался только у допубертатных детей, что продемонстрировало крайнюю важность раннего начала лечения [62–64].

Проведя эпианализ результатов 6-летней терапии соматропином 4 рандомизированных многоцентровых исследований, de Zegher F. с коллегами продемонстрировали больший ростовой эффект ГР при применении больших доз: увеличение SDS роста от -3,4 до -0,6 при дозе 0,033 мг/кг/сут; от -4,0 до -0,3 — при 0,067 мг/кг/сут [65]. Различий в ростовом ответе между детьми, имевшими и не имевшими ДГР, получено не было [64][66], что свидетельствует у детей с ЗВУР об отсутствии ассоциации между ростовым ответом и уровнями стимулированного в ходе провокационных тестов СТГ. В более поздних исследованиях получены более скромные результаты, но и они подтвердили эффективность лечения ГР у низкорослых детей с ЗВУР. Так, в опубликованном многоцентровом японском исследовании (2009–2018) за 4,29 года терапии соматропином удалось увеличить SDS роста от -2,03±0,77 до +0,85±0,72 [62].

Таким образом, доза соматропина является важнейшим предиктором эффективности ростового ответа, особенно в течение первого года лечения [67], однако при его длительном применении дозозависимый эффект становится не столь существенным [38][68].

Конечный рост при длительном применении гормона роста у детей с ЗВУР. Результатом длительного применения ГР явилась нормализация конечного роста у большинства низкорослых детей с ЗВУР [68]. Показано, что длительная терапия соматропином приводит к достижению конечного роста в пределах нормальных популяционных значений (>-2 SD) у 85% детей и в пределах генетически прогнозируемых значений — у 98%. SDS конечного роста в среднем составил в группе 1 -1,1±0,7 и в группе 2 — -0,9±0,8, в обеих группах наблюдалось значительное улучшение SDS роста по сравнению с исходными значениями, при этом достоверных различий по SDS конечного роста между группами получено не было, что свидетельствовало об эффективности обеих доз соматропина у большинства детей. SDS конечного роста в контрольной группе, не получавшей лечения (N=29, на момент начала исследования различий между контрольной и дозовыми группами не было), составил -2,3±0,7 и был достоверно ниже, чем в группах на лечении [68].

Эпианализ 4 рандомизированных многоцентровых исследований длительного, в течение 6 лет, применения соматропина показал среднее увеличение роста на 2,0 SD при дозе соматропина 0,033 мг/кг/сут и на 2,7 SD — при дозе 0,067 мг/кг/сут [65]. Данные о конечном росте на фоне длительной терапии соматропином у низкорослых детей с ЗВУР до сих пор остаются крайне ограниченными. Опубликованные результаты влияния терапии ГР на конечный рост у данной когорты детей суммированы в таблице 2.

**Table:** Таблица 2. Результаты конечного роста низкорослых детей с ЗВУР на фоне длительной терапии гормоном ростаTable 2. Outcomes of the final growth of short children with intrauterine growth retardation on the background of long-term therapy with growth hormone

	N	Доза гормона роста, мг/кг/сут	Возраст на момент начала терапии, годы	SDS роста на момент начала терапии	SDS конечного роста
Coutant R., et al., 1998 [69]	70	0,020	10,7±2,5	-2,9±0,8	-2,0±0,7
Zucchini S., et al., 2001 [70]	29	0,032	10,9±0,4	-2,3±0,1	-1,7±0,2
Van Pareren Y., et al., 2003 [68]	54	0,033 или 0,067	8,1±1,9	-3,0±0,7	-1,1±0,7 -0,9±0,8
Dahlgren J., et al., 2005 [71]	77	0,033	10,7±2,5	-2,8±0,7	-1,2±0,7
Rosilio M., et al., 2005 [72]	35	0,067	9,6±0,9	-2,6±0,5	-2,0±0,8
Tanaka T., et al., 2015 [44]	11	0,033/0,067	7,2 (м) 5,5 (ж)	-3,5 -3,5	-1,9 -1,7
Thomas M., et al., 2018 [73]	47	0,042±8,4	10,1±2,8	-3,2 ± 0,6	−2,1±1,0
Horikawa R., et al., 2020 [62]	32	0,248±0,068 (1-й год) 0,061±0,071 (2–7-й год)	10,8±2,1	-2,9±0,4	-2,0±0,8

Невысокая эффективность терапии ГР в некоторых исследованиях обусловлена небольшой длительностью терапии, перерывами в лечении либо низкими дозами соматропина.

Данные, полученные в ФГБУ «НМИЦ эндокринологии» Минздрава России, свидетельствуют о том, что при условии рано начатого, непрерывного, длительного (до достижения конечного роста, средняя продолжительность терапии 7,6±2,0 года) лечения ГР, конечный рост у мальчиков составляет 167,6±4,5 см (SDS конечного роста: 0,7±0,3), у девочек — 156,0±4,1 см (SDS конечного роста: 1,0±0,4), что значительно выше роста детей с ЗВУР без лечения: 158,0±3,9 см (SDS: -2,5±0,6) и 143,1±4,7 см (SDS: -3,2±0,8) соответственно [38]. За время лечения Δ между конечным и средне-родительским ростом удалось значимо сократить: до 5,2±1,5 см (SDS: 0,8±0,3) у мальчиков и до 6,2±2,1 см (SDS: 1,0±0,4) у девочек (исходно они составляли 16,0±4,3 см (SDS: 2,4±0,7) и 16,4±5,0 см (SDS: 2,8±1,0) соответственно). В контрольной группе, не получавшей лечения, уменьшения Δ между конечным достигнутым ростом и средне-родительским за аналогичное время наблюдения не произошло.

Успешность терапии соматропином зависит от ее продолжительности в допубертатном возрасте. Продемонстрировано, что Δ SDS роста у детей, получавших ГР более 2 лет до начала полового развития, составила 1,7 (12 см) по сравнению с 0,9 (6 см) у тех, кто до пубертата лечился менее 2 лет [62][71]. Множественный регрессионный анализ показал значительно меньшую эффективность лечения ГР у пубертатных (на момент начала лечения) детей, лиц женского пола, а также детей, которые намомент исследования имели меньшую массу тела [73]. Выявлена положительная корреляция между SDS роста на момент начала лечения и SDS конечного роста [38][73].

## ПРЕДИКТОРЫ РОСТОВОГО ОТВЕТА НА ТЕРАПИЮ ГОРМОНОМ РОСТА

К несомненным факторам, определяющим величину ростового ответа на терапию ГР, относятся: ХВ на момент начала терапии [38][59][65][67], доза соматропина [38][64][65][74], а также индивидуальный (относительно средне-родительского) дефицит роста [75].

По данным ФГБУ «НМИЦ эндокринологии» Минздрава России, эффективность терапии соматропином у низкорослых детей с ЗВУР положительно коррелирует с ростом биологических родителей, дозой соматропина и продолжительностью лечения; отрицательно — с хронологическим и костным возрастом на момент начала лечения, ХВ на момент начала пубертата [38].

Гормональные факторы. Скромные результаты лечения ГР низкорослых детей с ЗВУР, полученные в ряде исследований [65][75–77], послужили поводом для некоторых сомнений относительно ее эффективности у данной группы пациентов. Причина гетерогенного ростового эффекта до сих пор остается неясной [78]. Некоторыми авторами выдвинуто предположение о связи ростового ответа на соматропин с исходным уровнем ИФР1 [79] или спонтанными концентрациями ночного профиля СТГ, другими авторами связь исходных концентраций СТГ, ИФР1 и ИФРСБ3 с эффективностью лечения ГР не поддерживается [80].

Данные ФГБУ «НМИЦ эндокринологии» Минздрава России свидетельствует об отсутствии влияния на конечный рост значений СТГ на стимуляционной пробе и уровня ИФР1 на момент начала терапии [38].

Вероятность существования широкого спектра повреждений соматотропной оси у низкорослых детей с ЗВУР может объяснить гетерогенность ростового ответа на терапию ГР и необходимость индивидуального подхода к лечению этой категории детей.

## СКЕЛЕТНОЕ СОЗРЕВАНИЕ НА ФОНЕ ТЕРАПИИ ГОРМОНОМ РОСТА

Процессы линейного роста и костного созревания у детей тесно взаимосвязаны. Ускорение костного созревания может ограничить ростовой потенциал ребенка. Ранее было продемонстрировано ускорение костного созревания при лечении большими дозами ГР детей с идиопатической низкорослостью, в связи с чем вопрос о скорости костного созревания у детей с ЗВУР, получающих терапию соматропином, является крайне актуальным.

В нескольких исследованиях было показано, что у низкорослых детей с ЗВУР на фоне лечения ГР отсутствует ускорение костного созревания, скорость костного созревания в различных дозовых группах схожа [62][64][65][81]. Имеющиеся в литературе данные свидетельствуют в пользу того, что у низкорослых детей с ЗВУР терапия ГР не влияет на темпы естественного костного созревания [44][65]. Данные ФГБУ «НМИЦ эндокринологии» Минздрава России свидетельствуют о наличии ускорения костного созревания на фоне терапии ГР, темпы которого пропорциональны величине ускорения в росте: соотношение КВ/ХВ достоверно коррелирует с динамикой роста [38].

На фоне ростостимулирующей терапии ГР у низкорослых детей с ЗВУР наблюдается более раннее начало полового развития, у девочек — более раннее наступление менархе по сравнению с пациентами группы контроля, не получавшими лечения. Эффект носит дозозависимый характер. Ускоряя начало пубертата у данной когорты детей, терапия соматропином не влияет на продолжительность полового развития [38].

## УГЛЕВОДНЫЙ ОБМЕН НА ФОНЕ ТЕРАПИИ ГОРМОНОМ РОСТА

Хорошо известно, что ГР, являясь контринсулярным гормоном, может влиять на углеводный обмен, индуцируя инсулиновую резистентность.

На фоне терапии соматропином отмечается увеличение уровней глюкозы и гликированного гемоглобина, при этом их значения остаются в пределах нормы. Помимо этого, на фоне лечения соматропином наблюдается увеличение концентраций инсулина натощак, изменение уровней индексов: повышение HOMA-IR, снижение Caro и QUICKI, свидетельствующих о повышении инсулинорезистентности [79][82][83]. Нежелательное влияние ГР на углеводный обмен, встречающееся у низкорослых детей с ЗВУР, большинством исследователей признается как обратимое, после прекращения терапии соматропином показатели возвращаются к исходным значениям [62][66][79].

## ОНКОЛОГИЧЕСКИЕ РИСКИ НА ФОНЕ ТЕРАПИИ ГОРМОНОМ РОСТА

На сегодняшний день в литературе имеются единичные свидетельства того, что большая масса тела при рождении является независимым фактором риска неоплазий в частности — рака молочной железы [84]. Данных о влиянии низкой массы телана риск развития неоплазий, в настоящее время нет. Каждый факт появления онкологического заболевания на фоне применения гормона роста тщательным образом отслеживается. За все время применения терапии ГР не было получено достоверных данных об увеличении онкорисков у низкорослых детей с ЗВУР, тем не менее данный вопрос остается недостаточно изученным и крайне актуальным, в связи с чем требует дальнейших наблюдений.

## ДОПОЛНИТЕЛЬНАЯ ИНФОРМАЦИЯ

Источники финансирования. Работа выполнена по инициативе автора без привлечения финансирования.

Конфликт интересов. Автор декларируют отсутствие явных и потенциальных конфликтов интересов, связанных с публикацией настоящей статьи.

Участие авторов. Автор одобрил финальную версию статьи перед публикацией, выразил согласие нести ответственность за все аспекты работы, подразумевающую надлежащее изучение и решение вопросов, связанных с точностью или добросовестностью любой части работы.

## References

[cit1] Lee Peter A., Chernausek Steven D., Hokken-Koelega Anita C. S., Czernichow Paul (2004). International Small for Gestational Age Advisory Board Consensus Development Conference Statement: Management of Short Children Born Small for Gestational Age, April 24–October 1, 2001. Pediatrics.

[cit2] Clayton P. E., Cianfarani S., Czernichow P., Johannsson G., Rapaport R., Rogol A. (2007). Management of the Child Born Small for Gestational Age through to Adulthood: A Consensus Statement of the International Societies of Pediatric Endocrinology and the Growth Hormone Research Society. The Journal of Clinical Endocrinology & Metabolism.

[cit3] Karlberg J, Albertsson-Wikland K (2007). Growth in Full- Term Small-for-Gestational-Age Infants: From Birth to Final Height. Pediatric Research.

[cit4] Lee Anne CC, Katz Joanne, Blencowe Hannah, Cousens Simon, Kozuki Naoko, Vogel Joshua P, Adair Linda, Baqui Abdullah H, Bhutta Zulfiqar A, Caulfield Laura E, Christian Parul, Clarke Siân E, Ezzati Majid, Fawzi Wafaie, Gonzalez Rogelio, Huybregts Lieven, Kariuki Simon, Kolsteren Patrick, Lusingu John, Marchant Tanya, Merialdi Mario, Mongkolchati Aroonsri, Mullany Luke C, Ndirangu James, Newell Marie-Louise, Nien Jyh Kae, Osrin David, Roberfroid Dominique, Rosen Heather E, Sania Ayesha, Silveira Mariangela F, Tielsch James, Vaidya Anjana, Willey Barbara A, Lawn Joy E, Black Robert E (2013). National and regional estimates of term and preterm babies born small for gestational age in 138 low-income and middle-income countries in 2010. The Lancet Global Health.

[cit5] Tudehope David, Vento Maximo, Bhutta Zulfiqar, Pachi Paulo (2013). Nutritional Requirements and Feeding Recommendations for Small for Gestational Age Infants. The Journal of Pediatrics.

[cit6] Campisi Susan C, Carbone Sarah E, Zlotkin Stanley (2018). Catch-Up Growth in Full-Term Small for Gestational Age Infants: A Systematic Review. Advances in Nutrition.

[cit7] Chrestani Maria Aurora, Santos Iná S., Horta Bernardo L., Dumith Samuel C., de Oliveira Dode Maria Alice Souza (2012). Associated Factors for Accelerated Growth in Childhood: A Systematic Review. Maternal and Child Health Journal.

[cit8] Katz Joanne, Wu Lauren A., Mullany Luke C., Coles Christian L., Lee Anne C. C., Kozuki Naoko, Tielsch James M. (2014). Prevalence of Small-for-Gestational-Age and Its Mortality Risk Varies by Choice of Birth-Weight-for-Gestation Reference Population. PLoS ONE.

[cit9] de Onis Mercedes (2020). Update on the Implementation of the WHO Child Growth Standards. World Review of Nutrition and Dietetics.

[cit10] Villar José, Ismail Leila Cheikh, Victora Cesar G, Ohuma Eric O, Bertino Enrico, Altman Doug G, Lambert Ann, Papageorghiou Aris T, Carvalho Maria, Jaffer Yasmin A, Gravett Michael G, Purwar Manorama, Frederick Ihunnaya O, Noble Alison J, Pang Ruyan, Barros Fernando C, Chumlea Cameron, Bhutta Zulfiqar A, Kennedy Stephen H (2014). International standards for newborn weight, length, and head circumference by gestational age and sex: the Newborn Cross-Sectional Study of the INTERGROWTH-21st Project. The Lancet.

[cit11] Logvinova I.I., Emel'yanova A.S. Faktory riska rozhdeniya malovesnykh detei, struktura zabolevaemosti, smertnosti // Rossiiskii pediatricheskii zhurnal. — 2000. — №4. — S. 50-52

[cit12] Ross MG, Editor C, Smith C, et al. Fetal growth restriction. Obstetrics and gynecology [Internet]. [cited Sep 15, 2020]. Available from: https://emedicine.medscape.com/article/261226-overview

[cit13] Nardozza Luciano Marcondes Machado, Caetano Ana Carolina Rabachini, Zamarian Ana Cristina Perez, Mazzola Jaqueline Brandão, Silva Carolina Pacheco, Marçal Vivian Macedo Gomes, Lobo Thalita Frutuoso, Peixoto Alberto Borges, Araujo Júnior Edward (2017). Fetal growth restriction: current knowledge. Archives of Gynecology and Obstetrics.

[cit14] Stephenson T., Symonds M.E. Maternal nutrition as a determinant of birth weight. Archives of disease in childhood. Fetal and neonatal edition [Internet]. 2002;86(1). Available from: https://www.ncbi.nlm.nih.gov/pmc/articles/PMC1721347/pdf/v086p000F4.pdf 10.1136/fn.86.1.F4 PMC1721347 11815538

[cit15] Finken MJJ, van der Steen M, Smeets CCJ, et al. Children Born Small for Gestational Age: Differential Diagnosis, Molecular Genetic Evaluation, and Implications. Endocr Rev. 2018;39(6):851-894. doi: https://doi.org/10.1210/er.2018-00083 29982551

[cit16] Macdonald-Wallis C., Silverwood R. J., de Stavola B. L., Inskip H., Cooper C., Godfrey K. M., Crozier S., Fraser A., Nelson S. M., Lawlor D. A., Tilling K. (2015). Antenatal blood pressure for prediction of pre-eclampsia, preterm birth, and small for gestational age babies: development and validation in two general population cohorts. BMJ.

[cit17] Allen Victoria M, Joseph KS, Murphy Kellie E, Magee Laura A, Ohlsson Arne (2004). The effect of hypertensive disorders in pregnancy on small for gestational age and stillbirth: a population based study. BMC Pregnancy and Childbirth.

[cit18] Melchiorre Karen, Sutherland George Ross, Liberati Marco, Thilaganathan Basky (2012). Maternal Cardiovascular Impairment in Pregnancies Complicated by Severe Fetal Growth Restriction. Hypertension.

[cit19] AHLUWALIA I (2002). Multiple lifestyle and psychosocial risks and delivery of small for gestational age infants*1. Obstetrics & Gynecology.

[cit20] Anderson Ngaire H., Sadler Lynn C., Stewart Alistair W., Fyfe Elaine M., McCowan Lesley M.E. (2012). Independent risk factors for infants who are small for gestational age by customised birthweight centiles in a multi-ethnic New Zealand population. Australian and New Zealand Journal of Obstetrics and Gynaecology.

[cit21] Zhang Xun, Cnattingius Sven, Platt Robert W., Joseph K.S., Kramer Michael S. (2007). Are Babies Born to Short, Primiparous, or Thin Mothers “Normally” or “Abnormally” Small?. The Journal of Pediatrics.

[cit22] Woods KA, Camacho-Hübner C., Barter D., Clark AJL, Savage MO (2012). Insulin-like growth factor I gene deletion causing intrauterine growth retardation and severe short stature. Acta Paediatrica.

[cit23] Burton Graham J., Jauniaux Eric (2018). Pathophysiology of placental-derived fetal growth restriction. American Journal of Obstetrics and Gynecology.

[cit24] Townsend R., Khalil A. (2018). Fetal growth restriction in twins. Best Practice & Research Clinical Obstetrics & Gynaecology.

[cit25] Woods KA, Camacho-Hübner C., Barter D., Clark AJL, Savage MO (2012). Insulin-like growth factor I gene deletion causing intrauterine growth retardation and severe short stature. Acta Paediatrica.

[cit26] David Richard J., Collins James W. (2002). Differing Birth Weight among Infants of U.S.-Born Blacks, African-Born Blacks, and U.S.-Born Whites. New England Journal of Medicine.

[cit27] Strizhakov A.N., Timokhina E.V., Tarabrina T.V. Klinicheskoe znachenie insulinopodobnogo faktora pri sindrome zaderzhki razvitiya ploda // Voprosy ginekologii, akusherstva i perinatologii. — 2009. — T. 8. — №5. — S. 5-9.

[cit28] Gluckman P D (2014). Clinical review 68: The endocrine regulation of fetal growth in late gestation: the role of insulin-like growth factors.. The Journal of Clinical Endocrinology & Metabolism.

[cit29] Gluckman Peter D., Harding Jane E. (2009). The Physiology and Pathophysiology of Intrauterine Growth Retardation. Hormone Research.

[cit30] Yang Sei Won, Yu Jee Suk (2003). Relationship of insulin-like growth factor-I, insulin-like growth factor binding protein-3, insulin, growth hormone in cord blood and maternal factors with birth height and birthweight. Pediatrics International.

[cit31] Jaquet D., Leger J., Levy-Marchal C., Oury J. F., Czernichow P. (2014). Ontogeny of Leptin in Human Fetuses and Newborns: Effect of Intrauterine Growth Retardation on Serum Leptin Concentrations. The Journal of Clinical Endocrinology & Metabolism.

[cit32] Albertsson-Wikland Kerstin, Boguszewski Margaret, Karlberg Johan (2003). Children Born Small-for-Gestational Age: Postnatal Growth and Hormonal Status. Hormone Research in Paediatrics.

[cit33] Saenger P, Czernichow P, Hughes I, Reiter EO. Small for Gestational Age: Short Stature and Beyond. Endocr Rev. 2007;28(2):219-251. doi: https://doi.org/10.1210/er.2006-0039 17322454

[cit34] Ong KK, Dunger DB (2005). Birth weight, infant growth and insulin resistance. European Journal of Endocrinology.

[cit35] Campisi Susan C, Carbone Sarah E, Zlotkin Stanley (2018). Catch-Up Growth in Full-Term Small for Gestational Age Infants: A Systematic Review. Advances in Nutrition.

[cit36] Burlutskaya A.V., Shadrin S.A., Statova A.V. Fizicheskoe razvitie detei, rozhdennykh s zaderzhkoi vnutriutrobnogo razvitiya // Effektivnaya farmakoterapiya. — 2019. — T. 43. — №15. — S. 20-24. [Burlutskaya AV, Shadrin SA, Statova AV. Physical development of children born with developmental delay in utero. Effective pharmacotherapy. 2019;43(15):20-24 (In Russ.)]. doi: https://doi.org/10.33978/2307-3586-2019-15-43-20-24

[cit37] Leger Juliane, Limoni Constanzo, Collin Dominique, Czernichow Paul (2007). Prediction Factors in the Determination of Final Height in Subjects Born Small for Gestational Age. Pediatric Research.

[cit38] Nagaeva E.V. Rost, gormonal'nyi i metabolicheskii status u detei, rozhdennykh s zaderzhkoi vnutriutrobnogo razvitiya v raznye vozrastnye periody: Dis. … d-ra med. nauk — M.; 2021. 44 c.

[cit39] Waal W. J., Hokken-Koelega A. C. S., Stijnen Th., Muinck Keizer-Schrama S. M. P. F., Dropt S. L. S. (2008). Endogenous and stimulated GH secretion, urinary GH excretion, and plasma IGF-I and IGF-II levels in prepubertal children with short stature after intrauterine growth retardation. Clinical Endocrinology.

[cit40] Woods Katie A, Van Helvoirt Maria, Ong Ken K L, Mohn Angelica, Levy Jonathan, De Zegher Francis, Dunger David B (2007). The Somatotropic Axis in Short Children Born Small for Gestational Age: Relation to Insulin Resistance. Pediatric Research.

[cit41] Leger Juliane, Noel Michèle, Limal Jean Marie, Czernichow Paul (2007). Growth Factors and Intrauterine Growth Retardation. II. Serum Growth Hormone, Insulin-Like Growth Factor (IGF) I, and IGF-Binding Protein 3 Levels in Children with Intrauterine Growth Retar Age1. Pediatric Research.

[cit42] Arends N. J. T., Boonstra V. H., Duivenvoorden H. J., Hofman P. L., Cutfield W. S., Hokken-Koelega A. C. S. (2005). Reduced insulin sensitivity and the presence of cardiovascular risk factors in short prepubertal children born small for gestational age (SGA). Clinical Endocrinology.

[cit43] Ghirri P., Bernardini M., Vuerich M., Cuttano A. M. R., Coccoli L., Merusi I., Ciulli C., D'Accavio L., Bottone U., Boldrini A. (2009). Adrenarche ,pubertal development ,age at menarche and final height of full-term ,born small for gestational age (SGA) girls. Gynecological Endocrinology.

[cit44] Tanaka Toshiaki, Yokoya Susumu, Seino Yoshiki, Tada Hiroshi, Mishina Jun, Sato Takahiro, Hiro Shintaro, Ohki Nobuhiko (2015). Onset of puberty and near adult height in short children born small for gestational age and treated with GH: Interim analysis of up to 10 years of treatment in Japan. Clinical Pediatric Endocrinology.

[cit45] Verkauskiene Rasa, Petraitiene Indre, Albertsson Wikland Kerstin (2013). Puberty in Children Born Small for Gestational Age. Hormone Research in Paediatrics.

[cit46] Deng Xu, Li Wenyan, Luo Yan, Liu Shudan, Wen Yi, Liu Qin (2017). Association between Small Fetuses and Puberty Timing: A Systematic Review and Meta-Analysis. International Journal of Environmental Research and Public Health.

[cit47] Neville K A (2005). Precocious pubarche is associated with SGA, prematurity, weight gain, and obesity. Archives of Disease in Childhood.

[cit48] Milovanovic Ivana, Njuieyon Falucar, Deghmoun Samia, Chevenne Didier, Levy-Marchal Claire, Beltrand Jacques (2014). SGA Children with Moderate Catch-Up Growth Are Showing the Impaired Insulin Secretion at the Age of 4. PLoS ONE.

[cit49] Lithell H. O, McKeigue P. M, Berglund L., Mohsen R., Lithell U.-B., Leon D. A (2011). Relation of size at birth to non-insulin dependent diabetes and insulin concentrations in men aged 50-60 years. BMJ.

[cit50] Crispi Fatima, Crovetto Francesca, Gratacos Eduard (2018). Intrauterine growth restriction and later cardiovascular function. Early Human Development.

[cit51] Harder T., Rodekamp E., Schellong K., Dudenhausen J. W., Plagemann A. (2007). Birth Weight and Subsequent Risk of Type 2 Diabetes: A Meta-Analysis. American Journal of Epidemiology.

[cit52] Meas T., Deghmoun S., Alberti C., Carreira E., Armoogum P., Chevenne D., Lévy-Marchal C. (2010). Independent effects of weight gain and fetal programming on metabolic complications in adults born small for gestational age. Diabetologia.

[cit53] van Wassenaer A. Neurodevelopmental consequences of being born SGA. Pediatr Endocrinol Rev. 2005;2(3):372-377. 16429113

[cit54] Baud Olivier, Berkane Nadia (2019). Hormonal Changes Associated With Intra-Uterine Growth Restriction: Impact on the Developing Brain and Future Neurodevelopment. Frontiers in Endocrinology.

[cit55] Singh S. P., Ehmann S., Snyder A. K. (2013). Ethanol-Induced Changes in Insulin-Like Growth Factors and IGF Gene Expression in the Fetal Brain. Experimental Biology and Medicine.

[cit56] Strauss Richard S. (2003). Adult Functional Outcome of Those Born Small for Gestational Age. JAMA.

[cit57] Cianfarani Stefano, Maiorana Arianna, Geremia Caterina, Scirè Giuseppe, Spadoni Gian Luigi, Germani Daniela (2003). Blood Glucose Concentrations are Reduced in Children Born Small for Gestational Age (SGA), and Thyroid-Stimulating Hormone Levels are Increased in SGA with Blunted Postnatal Catch-up Growth. The Journal of Clinical Endocrinology & Metabolism.

[cit58] Cohen Pinchas, Bright George M., Rogol Alan D., Kappelgaard Anne-Marie, Rosenfeld Ron G. (2014). Effects of Dose and Gender on the Growth and Growth Factor Response to GH in GH-Deficient Children: Implications for Efficacy and Safety. The Journal of Clinical Endocrinology & Metabolism.

[cit59] Sas Theo, de Waal Wouter, Mulder Paul, Houdijk Mieke, Jansen Maarten, Reeser Maarten, Hokken-Koelega Anita (2014). Growth Hormone Treatment in Children with Short Stature Born Small for Gestational Age: 5-Year Results of a Randomized, Double-Blind, Dose-Response Trial^1^. The Journal of Clinical Endocrinology & Metabolism.

[cit60] Leger J., Levy-Marchal C., Bloch J., Pinet A., Chevenne D., Porquet D., Collin D., Czernichow P. (2011). Reduced final height and indications for insulin resistance in 20 year olds born small for gestational age: regional cohort study. BMJ.

[cit61] Carel Jean-Claude, Chatelain Pierre, Rochiccioli Pierre, Chaussain Jean-Louis (2003). Improvement in Adult Height after Growth Hormone Treatment in Adolescents with Short Stature Born Small for Gestational Age: Results of a Randomized Controlled Study. The Journal of Clinical Endocrinology & Metabolism.

[cit62] Horikawa Reiko, Tanaka Toshiaki, Nishinaga Hiromi, Nishiba Yosuke, Yokoya Susumu (2020). The long-term safety and effectiveness of growth hormone treatment in Japanese children with short stature born small for gestational age. Clinical Pediatric Endocrinology.

[cit63] Houk Christopher P, Lee Peter A (2012). Early diagnosis and treatment referral of children born small for gestational age without catch-up growth are critical for optimal growth outcomes. International Journal of Pediatric Endocrinology.

[cit64] Sas Theo C. J., Gerver Willem-Jan M., De Bruin Rob, Mulder Paul G. H., Cole Tim J., De Waal Wouter, Hokken-Koelega Anita C. S. (2003). Body proportions during 6 years of GH treatment in children with short stature born small for gestational age participating in a randomised, double-blind, dose-response trial*. Clinical Endocrinology.

[cit65] de Zegher Francis, Albertsson-Wikland Kerstin, Wollmann Hartmut A., Chatelain Pierre, Chaussain Jean-Louis, Löfström Annika, Jonsson Björn, Rosenfeld Ron G. (2014). Growth Hormone Treatment of Short Children Born Small for Gestational Age: Growth Responses with Continuous and Discontinuous Regimens Over 6 Years^1^. The Journal of Clinical Endocrinology & Metabolism.

[cit66] de Zegher Francis, Francois Inge, van Helvoirt Monique, Beckers Dominique, Ibáñez Lourdes, Chatelain Pierre (2002). Growth Hormone Treatment of Short Children Born Small for Gestational Age. Trends in Endocrinology & Metabolism.

[cit67] Ranke Michael B., Lindberg Anders (2008). Predicting growth in response to growth hormone treatment. Growth Hormone & IGF Research.

[cit68] Van Pareren Yvonne, Mulder Paul, Houdijk Mieke, Jansen Maarten, Reeser Maarten, Hokken-Koelega Anita (2003). Adult Height after Long-Term, Continuous Growth Hormone (GH) Treatment in Short Children Born Small for Gestational Age: Results of a Randomized, Double-Blind, Dose-Response GH Trial. The Journal of Clinical Endocrinology & Metabolism.

[cit69] Coutant Régis, Carel Jean-Claude, Letrait Muriel, Bouvattier Claire, Chatelain Pierre, Coste Joël, Chaussain Jean-Louis (2014). Short Stature Associated with Intrauterine Growth Retardation: Final Height of Untreated and Growth Hormone-Treated Children. The Journal of Clinical Endocrinology & Metabolism.

[cit70] Zucchini S (2002). Final height of short subjects of low birth weight with and without growth hormone treatment. Archives of Disease in Childhood.

[cit71] Dahlgren Jovanna, Wikland Kerstin Albertsson (2004). Final Height in Short Children Born Small for Gestational Age Treated with Growth Hormone. Pediatric Research.

[cit72] Rosilio Myriam, Carel Jean-Claude, Ecosse Emmanuel, Chaussainon Jean-Louis, _ _ (2005). Adult height of prepubertal short children born small for gestational age treated with GH. European Journal of Endocrinology.

[cit73] Thomas M., Beckers D., Brachet C., Dotremont H., Lebrethon M.-C., Lysy P., Massa G., Reynaert N., Rooman R., van der Straaten S., Roelants M., De Schepper J. (2018). Adult Height after Growth Hormone Treatment at Pubertal Onset in Short Adolescents Born Small for Gestational Age: Results from a Belgian Registry-Based Study. International Journal of Endocrinology.

[cit74] Ranke Michael B, Lindberg Anders (2011). Prediction models for short children born small for gestational age (SGA) covering the total growth phase. Analyses based on data from KIGS (Pfizer International Growth Database). BMC Medical Informatics and Decision Making.

[cit75] Fjellestad-Paulsen A, Czernichow P, Brauner R, et al. Three-year data from a comparative study with recombinant human growth hormone in the treatment of short stature in young children with intrauterine growth retardation. Acta Paediatr. 1998;87(5):511-517. doi: https://doi.org/10.1080/08035259850158209 9641731

[cit76] Tanaka Toshiaki, Yokoya Susumu, Seino Yoshiki, Togari Hajime, Mishina Jun, Kappelgaard Anne-Marie, Fujieda Kenji (2011). Long-Term Efficacy and Safety of Two Doses of Growth Hormone in Short Japanese Children Born Small for Gestational Age. Hormone Research in Paediatrics.

[cit77] Agut Thais, León Marisol, Rebollo Mónica, Muchart Jordi, Arca Gemma, Garcia-Alix Alfredo (2014). Early identification of brain injury in infants with hypoxic ischemic encephalopathy at high risk for severe impairments: accuracy of MRI performed in the first days of life. BMC Pediatrics.

[cit78] van der Steen Manouk, Pfundt Rolph, Maas Stephan J.W.H., Bakker-van Waarde Willie M., Odink Roelof J., Hokken-Koelega Anita C.S. (2016). ACAN Gene Mutations in Short Children Born SGA and Response to Growth Hormone Treatment. The Journal of Clinical Endocrinology & Metabolism.

[cit79] de Zegher Francis, Ong Ken, van Helvoirt Maria, Mohn Angelica, Woods Katie, Dunger David (2014). High-Dose Growth Hormone (GH) Treatment in Non-GH-Deficient Children Born Small for Gestational Age Induces Growth Responses Related to Pretreatment GH Secretion and Associated with a Reversible Decrease in Insulin Sensitivity. The Journal of Clinical Endocrinology & Metabolism.

[cit80] Leger Juliane, Noel Michèle, Limal Jean Marie, Czernichow Paul (2007). Growth Factors and Intrauterine Growth Retardation. II. Serum Growth Hormone, Insulin-Like Growth Factor (IGF) I, and IGF-Binding Protein 3 Levels in Children with Intrauterine Growth Retar Age1. Pediatric Research.

[cit81] Moon Jung-Eun, Ko Cheol Woo (2019). Delayed Bone Age Might Accelerate the Response to Human Growth Hormone Treatment in Small for Gestational Age Children with Short Stature. International Journal of Endocrinology.

[cit82] Cutfield Wayne S., Jackson Wendy E., Jefferies Craig, Robinson Elizabeth M., Breier Bernhard H., Richards Gail E., Hofman Paul L. (2003). Reduced insulin sensitivity during growth hormone therapy for short children born small for gestational age. The Journal of Pediatrics.

[cit83] Sas Theo, Mulder Paul, Aanstoot Henk Jan, Houdijk Mieke, Jansen Maarten, Reeser Maarten, Hokken-Koelega Anita (2003). Carbohydrate metabolism during long-term growth hormone treatment in children with short stature born small for gestational age. Clinical Endocrinology.

[cit84] AHLGREN M., MELBYE M., WOHLFAHRT J., SØRENSEN T.I.A. (2006). Growth patterns and the risk of breast cancer in women. International Journal of Gynecological Cancer.

